# Enablers and challenges to occupational therapists’ research engagement: A qualitative study

**DOI:** 10.1177/0308022617719218

**Published:** 2017-08-11

**Authors:** Laura Di Bona, Jennifer Wenborn, Becky Field, Sinéad M Hynes, Ritchard Ledgerd, Gail Mountain, Tom Swinson

**Affiliations:** 1 Engagement Manager/Occupational Therapist, Sheffield Health and Social Care NHS Foundation Trust, Sheffield, UK; 2 Honorary Research Fellow, School of Health and Related Research, 7315 University of Sheffield, Sheffield, UK; 3 Senior Clinical Research Associate/Occupational Therapist, Division of Psychiatry, University College London, London, UK; 4 Dementia Research Centre, Research and Development, North East London NHS Foundation Trust, London, UK; 5 Research Associate/Occupational Therapist, School of Health and Related Research, 7315 University of Sheffield, Sheffield, UK; 6 Lecturer, School of Medicine, Nursing and Health Sciences, 8799 National University of Ireland, Galway, Ireland; 7 Clinical Researcher/Occupational Therapist, Dementia Research Centre, North East London NHS Foundation Trust, London, UK; 8 Professor of Health Services Research, School of Health and Related Research, University of Sheffield, Sheffield, UK; 9 Senior Research Assistant, Dementia Research Centre, North East London NHS Foundation Trust, London, UK

**Keywords:** Occupational therapy, research, dementia

## Abstract

**Introduction:**

To develop occupational therapy’s evidence base and improve its clinical outcomes, occupational therapists must increase their research involvement. Barriers to research consumption and leadership are well documented, but those relating to delivering research interventions, less so. Yet, interventions need to be researched within practice to demonstrate their clinical effectiveness. This study aims to improve understanding of challenges and enablers experienced by occupational therapists who deliver interventions within research programmes.

**Method:**

Twenty-eight occupational therapists who participated in the Valuing Active Life in Dementia (VALID) research programme reported their experiences in five focus groups. Data were analysed thematically to identify key and subthemes.

**Results:**

Occupational therapists reported that overwhelming paperwork, use of videos, recruitment and introducing a new intervention challenged their research involvement, whereas support, protected time and a positive attitude enabled it. The impact of these challenges and enablers varied between therapists and organisations.

**Conclusion:**

Challenges and enablers to research involvement can be identified but must be addressed within individual and organisational contexts. Multifaceted collective action to minimise challenges and maximise enablers can facilitate clinicians’ involvement in research. Using this approach should enable occupational therapists to increase their research involvement, thus demonstrating the clinical effectiveness of their interventions.

## Introduction

Developing research-active clinicians and organisations has become a National Health Service (NHS) priority because of their positive impact on both clinical outcomes and the United Kingdom (UK) economy (Boaz et al., 2015; Department of Health (DH), 2006). For occupational therapists, incorporating research activity into their interventions and services is a requirement of their code of ethics and professional conduct (College of Occupational Therapists, 2015). When asked, the majority of occupational therapists report that they wish to be involved in research, but find it hard to do so, thus most are not ‘research active’ (Pighills et al., 2013; White et al., 2013). While this is the case, occupational therapists, alongside other allied health professionals in similar positions, risk being marginalised within healthcare delivery due to an inadequate evidence base (Pain et al., 2015).

There are many ways in which occupational therapists can engage with research. This has been termed a ‘research continuum’, ranging from activities that all occupational therapists should be doing, such as reading research literature, at one end, to research leadership activities, for the minority, at the other (Pighills et al., 2013). In England, the Department of Health’s ﬁve-year strategy for research and development, *Best Research for Best Health* (DH, 2006) led to increased investment in research infrastructure and capacity building for clinicians at all stages of this continuum. This included establishing the Health Education England/National Institute for Health Research (NIHR) clinical academic pathway to increase research capacity and leadership, and collaborations for leadership in applied health research and care (CLAHRCs) to increase applied research and its implementation (National Institute for Health Research, 2016a, 2016b). However, some research roles, such as healthcare professionals delivering research interventions and being research participants themselves, have received less focus despite the importance of this role for successful intervention development and research implementation (Hysong et al., 2013; Newall et al., 2009).

Occupational therapists have been integral to the success of a number of research studies by virtue of their role in delivering the interventions being investigated (for example Eriksson et al., 2013; Killaspy et al., 2015; Sturkenboom et al., 2014). The increasing research on occupational-therapist-delivered interventions is a real opportunity for the profession to extend its evidence base. However, there has been little consideration given to how to recruit and engage healthcare professionals as research participants and what might be the challenges and enablers to doing so (Hysong et al., 2013; Newall et al., 2009). The Valuing Active Life in Dementia (VALID) research programme involves occupational therapists in intervention delivery and has investigated the challenges and enablers to their engagement.

### The VALID research programme

The VALID research programme aims to develop and evaluate a community occupational therapy intervention for people living with mild to moderate dementia and their family carers: Community Occupational Therapy in Dementia–UK (COTiD-UK) (Wenborn et al., 2016). The programme builds on research of an intervention, community occupational therapy in dementia (COTiD), developed and found to be clinically and cost effective in the Netherlands (Graff et al., 2006, 2007, 2008). The VALID research programme follows the Medical Research Council’s framework for developing and evaluating complex interventions and as such consists of a number of phases, including a randomised controlled trial (Medical Research Council, 2008). The research reported in this paper was conducted within the initial multisite ‘development’ phase of the research programme, which customised the intervention to the UK setting and tested its feasibility in practice.

This development phase involved occupational therapists participating in a number of research activities (detailed in Table 1). Firstly, occupational therapists were trained in the COTiD intervention, (Graff et al., 2006, 2007). Secondly, they delivered this intervention to people living with dementia and their family carers. In some sites this also involved occupational therapists recruiting research participants and seeking informed consent. Finally, occupational therapists provided data about their activities within, and experiences of, the VALID research study. This included video recording intervention sessions, completing questionnaires about their own skill acquisition, detailing the time and content of the occupational therapy sessions provided and participating in a focus group.

**Table 1.** Occupational therapists’ research responsibilities.

Activity	Tasks
COTiD intervention training	• Attend five days’ training
	• Read additional training materials
	• Practice learning between sessions e.g. standardised assessments with volunteers (peers, students or service users)
	• Receive and reflect on written feedback from COTiD trainers about videoed sessions
	• Supervision from COTiD trainers (within VALID research team)
	• Peer supervision (frequency varied between sites)
Recruiting people living with dementia and their family carers as research participants to receive COTiD intervention (not all occupational therapists did this – in some sites, designated research staff completed this)	• Identify potential participants
	• Provide participant information sheets
	• Seek informed consent
	• Complete consent forms
	• Recruitment data management
COTiD intervention delivery	Provide 10 × 1 hour person-centred COTiD sessions in peoples’ homes/ community settings
Data collection	• Video record intervention sessions
	• Complete questionnaires about occupational therapists’ skill acquisition and transfer of knowledge into practice
	• Record date, duration and content of occupational therapy sessions
	• Discuss opinions in a focus group

This paper reports findings of focus groups in which occupational therapists discuss their involvement in the VALID research study, specifically what challenged or enabled their involvement.

### Study aim

To improve understanding of the challenges and enablers experienced by occupational therapists who deliver an intervention within a research study.

### Literature review: challenges and enablers to research participation

A number of studies have described the research challenges and enablers as experienced by occupational therapists internationally (for example Eriksson et al., 2013 in Sweden, Gutman, 2009 in the USA, Pighills et al., 2013 in Australia, White et al., 2013 in the UK). Also for other allied health professionals and nurses (for example Akerjordet et al., 2012 in Norway, McMaster et al., 2013 and Newall et al., 2009 in Australia). Three challenges to research involvement appear to be most frequently cited by occupational therapists and other healthcare professionals: lack of time, money and skills (Akerjordet et al., 2012; Gutman, 2009; McMaster et al., 2013; Pighills et al., 2013). Conversely, two enablers of research involvement are also widely cited: providing support and positive attitudes towards research (McMaster et al., 2013; Pain et al., 2015; Pighills et al., 2013; White et al., 2013). However, these studies have tended to focus on identifying general challenges and enablers to research engagement, often at unspecified stages of the research continuum.

In contrast, little has been documented about the experiences of healthcare professionals who get involved in delivering the intervention component of research. Only two, non-UK based, studies were identified: one of occupational therapists (Eriksson et al., 2013), the other of nurses (Newall et al., 2009), in both cases participating in randomised controlled trials. Both studies found that clinicians placed great value on taking part in research and that this motivation and the support of others acted as enablers to their participation. However, Eriksson et al. (2013) highlighted that a lack of time and/or experience and anxiety about skills were challenges to research participation. Both studies also identified that difficulties with research participant recruitment challenged their own involvement (Eriksson et al., 2013; Newall et al., 2009). Healthcare professionals, therefore, appear to report similar challenges and enablers to research engagement regardless of their profession or type of engagement along the research continuum. However, how these factors interact or how they apply to healthcare professionals delivering interventions within research programmes is not yet sufficiently understood.

## Method

### Design

Qualitative methods were selected as they are most appropriate for understanding participants’ experiences of little understood topics (Silverman, 2013), in this instance occupational therapists’ challenges and facilitators to research involvement. Qualitative methods can also facilitate deeper understanding of the contexts in which interventions will be delivered (Vernooij-Dassena and Moniz-Cook, 2014). Focus groups were chosen, in preference to interviews, to enable opinions to be gathered from more people and enable them to explore and clarify their views in a supportive environment (Kitzinger, 2000).

An indicative topic guide was developed by the research team aiming to elicit opinions on three topics; firstly, how the COTiD intervention should be adapted for the UK context; secondly, the most and least effective elements of the training provided for delivering the intervention; and thirdly, enablers and challenges to research participation. Data about the first two topics informed the development of the COTiD-UK intervention and training and will be reported separately. Data collected in relation to the last topic were used when planning the next phases of the VALID research programme and are reported here.

### Recruitment

Ten English healthcare organisations participated in the development phase of the VALID research programme, which included these focus groups (Wenborn et al., 2016). Each organisation was asked to identify occupational therapists who could participate in the study. Forty-four occupational therapists participated, and all met the eligibility criteria of being registered as an occupational therapist with the Health and Care Professions Council, with experience of working in the community and/or with people living with dementia and their family carers. All were invited to attend a focus group after they had completed the COTiD training and delivered the intervention.

### Ethical issues

The study was approved by the Yorkshire and the Humber – Leeds West National Health Service (NHS) Ethics Committee (reference number 12/YH/0492) on 16 November 2012. The study was also granted NHS research and development approval (reference number 13762). Occupational therapists provided signed informed consent. Data were anonymised and stored securely following usual data management procedures.

### Participants

Twenty-eight (64%) occupational therapists from eight (80%) of the participating healthcare organisations took part in focus groups, with between two and eight occupational therapists representing each organisation. Twenty-six (93%) were women. Seven (25%) were band five (junior occupational therapists, 13 (46%) band six (specialist occupational therapist), six (21%) band seven (highly specialist occupational therapist) and two (7%) band eight (lead occupational therapist).

### Data collection

Five focus groups were conducted with between five and eight occupational therapists attending each. Four members of the research team facilitated the focus groups, two facilitating each, with the exception of one smaller group with just three attendees that was facilitated by one person. The groups were audio recorded. Facilitators completed observational notes and reflexive analysis during and immediately following focus groups to document additional key information, such as participants’ facial expressions, gestures and researchers’ thoughts and interpretations. Audio recordings were independently transcribed verbatim and anonymised. Transcripts were checked for accuracy and missing data by focus group facilitators. As it was not possible to identify all focus group participants from the recorded transcript, each was identified only as a facilitator or participant.

### Data analysis

Thematic analysis was carried out (Guest et al., 2012). This involved each researcher reading one or two transcripts, ascribing codes, categories and then themes to the data. The research team discussed and iteratively checked these against the transcripts, looking for evidence of themes, categories and codes being confirmed or disconfirmed, to ensure trustworthiness and credibility (Mays and Pope, 2000). Once there was agreement on the overall themes, categories and codes were then reapplied to the transcripts.

## Results

**Figure 1.**
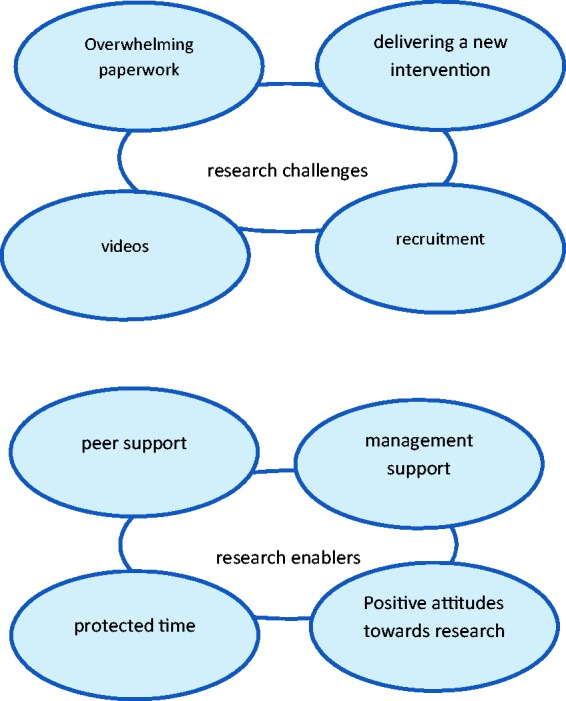
Occupational therapists’ research engagement: challenges and enablers.

Two main themes emerged describing occupational therapists’ ‘research challenges’ and ‘research enablers’, each with four subthemes. See Figure 1 for details.

### Research challenges

Within the theme of research challenges, four subthemes were identified: ‘overwhelming paperwork’, ‘videos’, ‘recruitment’ and ‘delivering a new intervention’. These topics were raised by participants in all focus groups.

#### Overwhelming paperwork

There was a general consensus that research involvement had led to engagement in a range of additional administration and reporting tasks. For some, paperwork and emails presented a real challenge both in terms of volume and because these were updated during the course of their involvement. One therapist stated:

I have felt so overwhelmed, I have actually just dumped the whole lot and done nothing. I am just so confused and all the changes … (Focus group 2 participant).

However, whilet many appeared to agree with this sentiment, for others, additional administrative tasks were accepted as an essential, if time consuming, part of the research process.

#### Videos

The occupational therapists were required to video record the intervention sessions and then transfer the videos to the research team via an encrypted USB memory stick. Many described this as challenging, although different aspects were raised in different focus groups. Firstly, some had never used video recorders before, so had to learn this new skill, which not everyone found easy. Secondly, the quality of the video recorders was criticised for having limited battery life and being difficult to position in order to get all three participants in view (person living with dementia, carer and occupational therapist). One therapist, who had encountered many challenges, stated:

We spent countless, I cannot tell you how many frustrating countless hours with these video recorders that don’t video, that don’t charge, that don’t do this (Focus group 4 participant).

Thirdly, the impact of the cameras on rapport building was viewed by some as a good ice-breaker but more often they were viewed negatively. For instance:

When the camera goes off as well … everything comes out (Focus group 4 participant).

A number of occupational therapists described this phenomena of ‘opening up’ when cameras had been switched off, as this was usually when the therapist was due to leave, resulting in increased time pressures as they stayed to listen to what people had held back from saying on camera. Finally, therapists described difficulties in transferring videos from the camera on to computers to view them and then onto USB memory sticks to send to the research team. In some cases, this was because of a lack of familiarity and skills with the technology, in other cases because their organisations required special permission to enable them to use the software provided or had inadequate hardware.

#### Recruitment

Three recruitment-related challenges were reported. Firstly, those responsible for recruiting people living with dementia and their family carer to receive the COTiD intervention stated this was time consuming and required additional paperwork to be completed.

Recruitment … there was a lot of phone calls and visits before you actually get someone to say yes please … so juggling that with everything else (Focus group 3 participant).

Secondly, the plan of having service users recruited in readiness for occupational therapists to start working with alongside attending the COTiD training did not work out for a variety of reasons across the organisations. This meant that some therapists had a delay between completing their COTiD training and having service users ready to work with. One therapist summed up the difficulties this caused:

I suspect if I had done the couple straight away … I would have remembered a bit more … been a bit more enthusiastic (Focus group 5 participant).

Finally, some therapists reported that the service users recruited were not appropriate for the intervention, making it harder to deliver. For instance, stating they had volunteered altruistically for the study rather than because they wanted or were appropriate to engage with the intervention.

#### Delivering a new intervention

Occupational therapists had to learn to deliver a new intervention, COTiD. They expressed inconsistent, different opinions on the intervention aims and design; for example, whether or not it had the right amount of structure, was the right length or was person-centred enough. However, the majority appeared to appreciate working in an occupational therapy profession-specific way, for example:

It really does give you an opportunity to go back to the core of OT and spend some quality time (Focus group 3 participants).

For most, working in ways that were different to routine practice was challenging:

With the way memory services work we go in with a plan and we do it and we go out so this has been quite difficult to take a step back (Focus group 5 participant).

For some, the necessary level of scrutiny of their work to meet the research protocol requirements was also quite challenging:

Well I felt I was going to get slated as soon as that film went to someone to watch … you’re in the fear of have you done wrong (Focus group 4 participant).

### Research enablers

Within the theme of research enablers were four subthemes, ‘peer support’, ‘management support’, ‘protected time’ and ‘positive attitudes towards research’. Each subtheme was identified in every focus group, with occupational therapy participants initiating discussion of the topic.

#### Peer support

All occupational therapists participated in peer support groups where they shared their experiences of participating in VALID, most also supported each other outside of these groups. They described three mechanisms by which this support enabled their research involvement. Firstly, by creating a safe environment for occupational therapists to practise clinical skills, such as standardised interviews, which they might not otherwise be using in day to day practice. Secondly, it enabled them to share feelings about research involvement and its challenges. Finally, it was useful to clarify quickly the required research procedures. For instance:

… somewhere to ask questions, like oh my goodness what do I do with this one rather than me ringing XX (lead researcher) (Focus group 1 participant).

While all who expressed an opinion described peer support as a positive enabler of research involvement, practical challenges to ensuring it happened were identified. Firstly, if there were not many occupational therapists within a geographical location, they either had to travel a long way for peer supervision or have it with such a small group that there were fewer experiences, views and expertise to be shared. However, if there were more therapists involved, finding a mutually convenient time and location to meet was difficult.

#### Management support

Occupational therapists reported differing experiences of management support. While all had management permission to engage in the study, some described additional mechanisms by which managers had facilitated their research involvement. Firstly, by championing VALID within their organisation, highlighting its importance to other colleagues and managers and encouraging occupational therapists to prioritise it within their workload. Secondly, by negotiating specific time, such as a day or two a week away from their usual role to focus solely on VALID. Finally, linking therapists with their research and development departments, who had subsequently provided further support, for instance with recruitment of participants and using the video cameras and associated software and hardware. One occupational therapist stated:

We couldn’t have done it without xx our OT (lead)… the amount of work that she has done (Focus group 3 participant).

In contrast to these positive experiences, some participants described having multiple managers with differing levels of enthusiasm and support for the research. Where support was more ambivalent, research involvement became more challenging. One occupational therapist stated:

Managers need to be on board… it was as if I was going off doing my own thing having a good time (Focus group 2 participant).

Occupational therapists, therefore, described how management support enabled research involvement in multifaceted ways, but highlighted that they often had more than one manager and it was easier when all were actively supportive.

#### Protected time

All occupational therapists who expressed an opinion stated that having protected, funded time, to focus solely on VALID was a major enabler of research involvement. In contrast, those participating in VALID without allowances made for their usual roles, described it causing tension within teams, as they were less available for other work. One therapist described it as ‘balancing two jobs’ [Focus group 1 participant], and some described feeling that this compromised the quality of both their clinical and research work. Most of those without protected time reported completing much of their research paperwork, intervention preparation and sometimes even the intervention, in their own time. Generally, risk management, crisis and generic work took priority. For instance:

By the time I got back to the office and you’ve got a semi crisis on VALID’s gone out the window (Focus group 4 participant).

In contrast, the minority who had protected time, such as 1 or 2 days a week focusing solely on VALID, did not describe the same difficulties.

#### Positive attitudes towards research

Occupational therapists mostly expressed positive attitudes towards research involvement. Some spoke with great enthusiasm about how participating in research had given them the opportunity to deliver an intervention they valued. COTiD was described positively by many as being ‘core OT’ and by some as person-centred and less time pressurised than much of their other work. One participant stated:

I was having this discussion with my manager, he said to me, ‘what is different?’ and I said ‘what is different is the quality’ (Focus group 3 participant).

For others, it was involvement in the research process itself that they valued, describing benefits for themselves as increasing their research understanding, capacity and experience. Some felt that by participating in VALID they were contributing to the development of the occupational therapy profession on a local and national level.

On a local level it’s very exciting for us to have this research to try and raise the profile of OT so you know I think it’s a fantastic opportunity to be part of it (Focus group 5 participant).

## Discussion

Occupational therapists reported that overwhelming paperwork, use of videos, recruitment and introducing a new intervention challenged their research involvement, whereas support, protected time and positive attitudes enabled it. These enablers and challenges broadly concur with those identified in previous studies of clinicians’ participation in randomised controlled trials (Eriksson et al., 2013; Newall et al., 2009). They are also similar to those identified at other stages of the research continuum, such as implementing evidence-based practice and research leadership development (Akerjordet et al., 2012; Gutman 2009; McMaster et al., 2013; Pain et al., 2015; Pighills et al., 2013; White et al., 2013).

Implementing a research study within clinical practice was found to be a complex, multistage process. While there were commonalities in the research challenges and enablers identified by occupational therapists, the degree of their impact varied. This is consistent with understandings from implementation science that individual and organisational contexts are hugely influential, to the extent that what one person considers a challenge, another may consider an enabler (Damschroder et al., 2009; May et al., 2016). Normalisation process theory helps explain how new practices are operationalised in healthcare and other settings (May and Finch, 2009). It describes four mechanisms through which changes to practice are implemented (May and Finch, 2009):

1. coherence (sense making and meaning);
2. cognitive participation (personal engagement);
3. collective action (organisational engagement and interaction to implement);
4. reflexive monitoring (reflection and appraisal).

This offers a useful way to consider how the challenges and enablers identified impacted on occupational therapists in this study.

### Attitudinal challenges and enablers

Occupational therapists were required to change their practice by implementing COTiD, a new intervention, and adhering to VALID research procedures. Consistent with previous research, most described this as challenging, despite contrasting opinions about the intervention and research involvement (Damschroder et al., 2009; Eriksson et al., 2013; May and Finch, 2009). Normalisation process theory explains that changes to practice are more likely to be adopted and viewed positively when individuals have coherence with them, i.e. new processes make sense to them (May and Finch, 2009). Coherence varied between occupational therapists, perhaps in relation to how similar COTiD and the research procedures were to their usual practice, therefore how large the changes were, and how much they approved of the changes. For instance, some who usually provided short-term or more prescriptive interventions appeared to relish the opportunity to work in a more person-centred way, while others did not. Occupational therapists who appeared to have coherence with the research and intervention spoke positively about being involved in VALID, consistent with previous findings that positive attitudes towards research facilitate engagement (Eriksson et al., 2013; Pain et al., 2015; Pighills et al, 2013; White et al, 2013).

### Practical challenges

Occupational therapists reported being challenged by recruitment, overwhelming paperwork and using videos. Recruiting service user participants was described as time consuming and challenging, consistent with previous findings (Hysong et al., 2013; Newall et al., 2009; Newington and Metcalfe, 2014). The importance of recruiting research participants to coincide with the readiness of therapists to work with them has been reported previously (Gitlin et al., 2010), and this current study highlights the potentially negative impact on therapists’ motivation when it does not happen in a timely manner. Using videos and completing research paperwork presented challenges for many reasons, varying between occupational therapists. These included familiarity and confidence with using videos, organising and completing paperwork, compatibility of data transfer with organisational systems and occupational therapists’ and service users’ attitudes to being videoed. Learning new skills has often been described as challenging to research involvement, although usually with reference to research skills (Akerjordet et al., 2012; McMaster et al., 2013; Pain et al., 2015), although problems using software have also been described previously (Damschroder et al., 2009). Cognitive participation may have influenced the impact of these challenges, in that some occupational therapists who were more personally motivated to engage in the research were perhaps happier to invest more time and energy into learning the necessary skills and processes. Conversely, it may also be that occupational therapists who encountered fewer challenges more quickly gained coherence and this facilitated cognitive participation with the research.

### Enablers

Peer and management support and protected time were identified as enablers, reducing the impact of challenges, consistent with previous findings (Eriksson et al., 2013; McMaster et al., 2013; Pain et al., 2015; Pighills et al., 2013; White et al., 2013). The mechanisms by which peer support enables research involvement have not previously been well described, but concur with previous reports that it is valued (Eriksson et al., 2013; Gitlin et al., 2010). This study’s findings suggest peer support enables research involvement by increasing confidence through creating a safe environment for practising clinical skills, sharing thoughts and feelings about research engagement and clarifying research expectations.

Management support was identified as a key multifaceted enabler, consistent with previous research (Newall et al., 2009; Pain et al., 2015; Perry et al., 2008; Pighills et al., 2013) . Management support provided: permission and encouragement for research involvement; assistance in negotiating with information technology departments to resolve software and hardware difficulties; support to negotiate with team members expressing negative attitudes towards research involvement; links to research and development departments to assist with recruitment and paperwork; and in some cases negotiating protected time for occupational therapists to engage in research. Protected time, with reduced clinical caseloads or time away from a usual clinical base, appeared an effective way to minimise clinical pressures, meaning that responding to crises was less likely to take priority over research activity, consistent with previous findings (Newall et al., 2009). When occupational therapists had more time allocated to their VALID work they used it to learn the new intervention and attend to paperwork, therefore diminishing the impact of these challenges, which may have positively affected their attitude to research. Normalisation process theory explains these enablers through collective action as organisations demonstrated their support for research involvement, minimised additional burdens on occupational therapists and positively influenced their sense of coherence and cognitive participation with the research. The impact of reflexive monitoring was less evident, perhaps due to the relatively short-term nature of therapists’ research involvement or because occupational therapists engaged in reflective monitoring within the focus groups.

### Implications

The consistency with which enablers and challenges to research are reported suggests they can be pre-empted but that their impact will vary between organisations and individuals. Therefore, while engaging clinicians in research is challenging, research programmes should try to ensure participating clinicians have access to protected research time, peer and management support. Positive attitudes to research should be encouraged and additional research tasks, such as recruiting participants, videoing and paperwork should be minimised. A greater understanding of the individual and organisational contexts in which occupational therapists work is required to assess the likely impact of challenges and enablers. It is, therefore, important to engage all stakeholders, service users, clinicians and managers, during the research planning phase to identify and minimise challenges and maximise the use of enablers (Eriksson et al., 2013; May and Finch, 2009). As well as helping practically, doing so may help to foster collective action, increase coherence and cognitive participation with the research, thus increasing the probability of successful implementation.

More in-depth study of enablers and challenges to delivering interventions as part of research studies would be beneficial to increase understanding about their impact, how they interact and vary between professional groups, organisational contexts or research designs. This could be achieved by using methods which gather more in-depth data, or data from a greater number or wider variety of participants. For instance, the themes arising in this study could be used as the basis for wider investigation such as through a quantitative semistructured survey or qualitative interviews.

### VALID research programme’s response to identified challenges and enablers

In the next stages of the VALID programme a number of steps were taken to enable occupational therapists’ research involvement. These included replacing video recording the intervention sessions with audio recording, minimising amendments to research paperwork and procedures, and allocating more time to practising use of hardware and software and related procedures within the training. Also, managers were invited to attend the training so as to understand better the requirements, dedicated research staff took responsibility for recruiting and consenting research participants and pre-trial checks were completed to guide research sites towards facilitating clinician involvement. Future publications will report on the impact of these changes.

### Limitations

As a focus group study it is not possible to generalise from these findings. It is not known whether these findings reflect the views of the other occupational therapists who participated in the VALID research programme but declined to participate in the focus groups, or whether they are representative of other occupational therapists with similar research experiences. As the focus groups were multipurpose, they may not have captured the full extent of opinions on research involvement. Finally, because transcription was outsourced it was not possible to be confident in ascribing quotations to individual participants.

## Conclusion

This study identified that while involving occupational therapists in research is challenging, it can be enabled by providing support, protected time and encouraging positive attitudes. While specific challenges and enablers were highlighted, which were broadly consistent with previous research, the need to consider them within individual and organisational contexts was also highlighted. Therefore, to enable occupational therapists’ involvement in research, multifaceted collective action involving all stakeholders is required to minimise challenges and maximise enablers. It is important to overcome challenges to research involvement so that occupational therapists can contribute to research alongside clinical practice, in line with their code of ethics and professional practice (College of Occupational Therapists, 2015). Also, to demonstrate the clinical effectiveness of their interventions, increase the occupational therapy evidence base and improve outcomes and experiences for service users.

## Key findings

Occupational therapists’ research involvement is challenged by implementing research tasks and new interventions but enabled by support, protected time and positive attitudes. Contexts vary the impact of challenges and enablers.

## What the study has added

This study has increased understanding of the challenges and enablers of engaging occupational therapists in research, through delivering interventions within research studies.
